# Rapidly evolving aphid gall effector proteins exhibit saposin-like folds

**DOI:** 10.1073/pnas.2609639123

**Published:** 2026-09-02

**Authors:** Fatema Bhinderwala, Aishwarya Korgaonkar, Kota Gopalakrishna, Thomas C. Mathers, Shuji Shigenobu, J. Fernando Bazan, Saskia A. Hogenhout, Guillermo Calero, Angela M. Gronenborn, David L. Stern

**Affiliations:** ^a^ Department of Structural Biology, University of Pittsburgh School of Medicine, Pittsburgh, PA 15261; ^b^ Janelia Research Campus, Janelia Research Campus, HHMI, Ashburn, VA 20147; ^c^HHMI, Kansas City, MO 64110; ^d^ Stowers Institute for Medical Research, Kansas City, MO 64110; ^e^ Department of Crop Genetics, John Innes Centre, Norwich NR4 7UH, United Kingdom; ^f^ Tree of Life, Wellcome Sanger Institute, Hinxton, Cambridge CB10 1SA, United Kingdom; ^g^ Laboratory of Evolutionary Genomics, Trans-Scale Biology Center, National Institute for Basic Biology, Myodaiji, Okazaki 444-8585, Japan; ^h^ℏ Bioconsulting llc, Stillwater, MN 55082

**Keywords:** protein structure, evolution, host-parasite, effector proteins, Alphafold

## Abstract

Many insects manipulate plants by injecting effector proteins. In one extreme example of this molecular “hijacking,” *Hormaphis cornu* aphids inject bicycle proteins into *Hamamelis virginiana*, contributing to the development of novel organs called galls. Bicycle proteins share no amino acid sequence similarity with proteins of known function. Here, we report the crystal structures of two divergent bicycle proteins. Both proteins contain saposin-like folds: one with multiple disulfide bonds exhibits a swapped domain topology; the other has no disulfide bonds and possesses two distinct, tandem domains. To explore the structural evolution of bicycle proteins, we attempted to predict bicycle protein structures with Alphafold2 (AF2) and other deep learning programs. While AF2 did not recover the two experimental structures using existing databases, it succeeded when provided with multiple sequence alignments (MSAs) of protein sequences from newly sequenced closely related species. Using this approach, we generated 2,400 high-confidence bicycle protein predictions from seven aphid species. While all aphid bicycle proteins contain predicted saposin-like folds, they display a vast diversity of structural and physicochemical properties. While this diversity thwarts prediction of conserved functions encoded in structure, it suggests that bicycle proteins have evolved to target diverse plant processes and/or to evade plant immune surveillance. Our extension of AF2 with custom MSAs of proteins from closely related species provides a generalizable, powerful approach for predicting structures of rapidly evolving protein families.

## Significance

Parasites introduce specialized “effector” proteins into hosts to suppress host immunity and to release nutrients. The molecular functions and structures of most effector proteins are unknown. Effector proteins often evolve rapidly and share no similarity with proteins of known function. Here, we demonstrate that machine learning algorithms can predict the structures of aphid “bicycle” effector proteins when supplemented with data from closely related species. We exploit this finding to generate predictions of 2,400 bicycle protein structures. Aphid bicycle proteins exploit a common folding motif, yet exhibit topologically distinct structures that form separate structural clusters. Despite the clustering of these proteins in structure space, they occupy a nearly uniform physicochemical space, suggesting that they encode a large diversity of molecular functions.

Pathogens employ effector proteins to manipulate host physiology, development, and behavior (1–6). In response to these effectors, hosts deploy immune responses. Over evolutionary time, conflict between effectors and immune responses can result in a so-called molecular “arms-race” between the host and pathogen, associated with rapid molecular evolution (7–11). This results in the generation of novel protein families that share little amino acid sequence similarity with proteins of known function, complicating the inference of their molecular functions from sequence-based comparisons.

Aphids are plant sap-sucking insects that secrete salivary proteins into plant cells to facilitate access to nutrients and inhibit plant defense responses (12). The most extreme examples of plant manipulation by aphids and other insects is the production of novel organs, called galls, which reflect reprogramming of plant cell growth and development (13, 14). Aphids and other insects induce galls to provide nutritious enclosed spaces that protect the insects from predators, parasites, and environmental vicissitudes (15–17). Bicycle proteins from *Hormaphis cornu* have recently been implicated as aphid-produced effectors that influence gall development (13). In particular, the bicycle protein Determinant of Gall Color specifically regulates a single anthocyanin biosynthetic pathway by suppressing pigment-related gene expression to turn red galls green (13). It is not currently clear how individual bicycle proteins perform such potent and specific manipulations of plant biology. *Bicycle* genes have evolved rapidly owing to strong diversifying selection (13), and the genome of the gall-inducing aphid *H. cornu* encodes more than 650 *bicycle* paralogs. All aphid genomes examined to date encode *bicycle* genes (18), suggesting that many or all aphids inject bicycle proteins into plants to manipulate plant physiology and development.

Bicycle proteins possess an N-terminal secretion signal sequence, which is likely removed during translocation to the secretory vesicles. A pair of distributed cysteine–tyrosine–cysteine motifs (C-Y-C) is found in most bicycle proteins—giving the family its name (“bi-CYC-like”)—and suggests that these proteins contain two structurally related domains. However, outside of the signal sequences, these proteins display no similarity to proteins or domains of known function (13). Here, we report the X-ray structures of two bicycle proteins from *H. cornu*. Both structures contain saposin-like folds, but in topologically different arrangements. These experimentally determined structures were used as positive controls to benchmark AlphaFold2 (AF2) predictions of additional bicycle proteins. Multiple sequence alignments (MSA) of bicycle proteins encoded by newly sequenced genomes of species closely related to *H. cornu* allowed AF2 to predict models that closely resembled the crystal structures, as well as high-confidence models for thousands of bicycle proteins from multiple species. These predicted models exhibit extensive variability in overall amino acid sequence space, especially of surface residues, as well as of physicochemical properties, with no evidence of conserved surface accessible domains that might define biochemical functions. The extensive diversity of these proteins may be required to allow them to engage with multiple molecular targets in plants and/or to evade plant defense mechanisms.

## Results

### A Bicycle Protein with Helix Swapped Saposin-Like Folds.

We selected 18 *bicycle* genes (*SI Appendix*, Table S1) that are highly expressed in *H. cornu* salivary glands based on RNA Sequencing (RNA-seq) data (13) for heterologous expression. Of these, four proteins expressed well in *Escherichia coli* and three in *SF9* insect cells and were purified in sufficient quantities for initial crystallization tests. Of these, two proteins—g3873 and g2703—yielded diffraction-quality crystals (*SI Appendix*, Fig. S1 and Table S1).

**Fig. 1.**
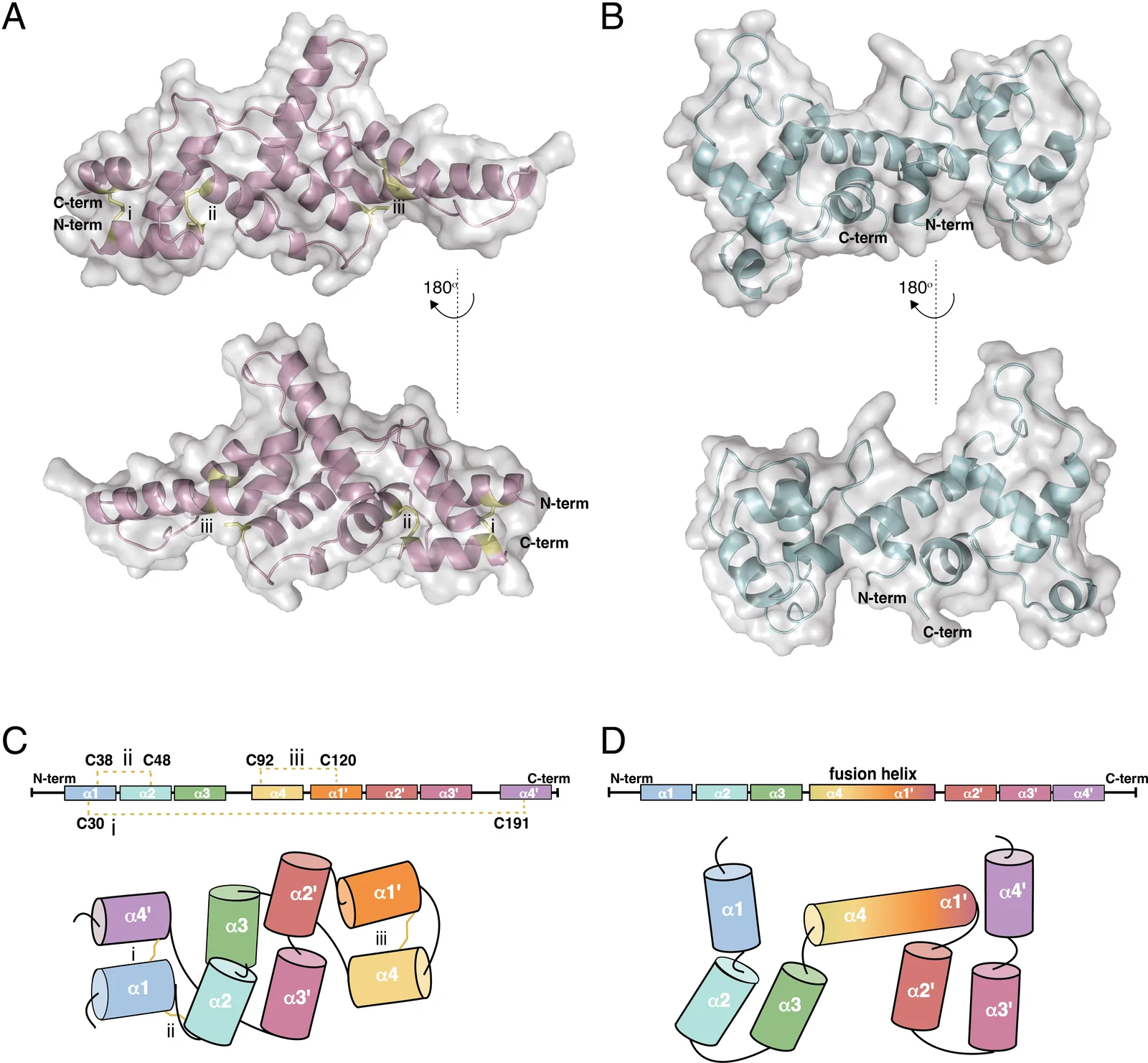
X-ray structures of two *H. cornu* bicycle proteins. (*A*) 2.0 Å X-ray structure of G3873 in ribbon representation (pink) with the three disulfide bonds and the free cysteine residue shown in stick representation (yellow), embedded in a gray surface representation. (*B*) 1.4 Å X-ray structure of G2703 in ribbon representation (aqua) embedded in a gray surface representation. (*C* and *D*) Schematic representations of the secondary structure elements in the G3873 (*C*) and G2703 (*D*) polypeptide chains. Helical elements are represented by colored boxes or cylinders, labeled α1 through α4’. In G3873, the left and right side four-helical bundles include α1, α2, α3, and α4’ and α1’, α2’, α3’, and α4 helices, respectively. In G2703, the left- and right-side four-helical bundles include α1, α2, α3, and the α4 portion of the α4-α1’ fused long central helix, and the α1’ portion of the fused long central helix, α2’, α3’, and α4’ helices, respectively. Color coding runs from the N terminus (aqua blue) to the C-terminus (purple). Disulfide bonds are indicated by yellow lines (dashed; *Top*; zig-zag *Bottom*).

The X-ray structure of g3873 was solved at 2.0 Å resolution using sulfur-SAD phasing and revealed an all-helical fold, possessing two subdomains, each comprising four-helical bundles: helices α1, α2, α3, and α4’ form one bundle, and α1’, α2’, α3’, and α4 form the second bundle (Fig. 1 *A* and *C* and *SI Appendix*, Fig. S2 and Table S2). The first (C30) and second (C191) conserved cysteines of the first and second CYC motif, respectively, form a disulfide bond linking helices α1 and α4’. The second (C92) and first (C120) conserved cysteines of the first and second CYC motif, respectively, form a second disulfide bond linking helices α4 and α1’ (Fig. 1*A*). A third disulfide bond connects C38 in helix α1 and C48 in helix α2, and these cysteines are not strongly conserved across bicycle proteins. The average distance between the sulfur atoms in the C30-C191, C92-C120, and C38-C48 bonds is 2.05 ± 0.1 Å, 2.08 ± 0.1 Å, and 2.03 ± 0.05 Å, respectively. The presence of three disulfide bonds as well as an additional free sulfhydryl group was confirmed by titration using 5,5′-dithiobis-2-nitrobenzoic acid (19, 20) and the sulfur positions were clearly identified in the difference density maps (*SI Appendix*, Fig. S3). The two disulfide bonds associated with the CYC motifs form left-handed (LH) spirals, with χ1 and χ1′ dihedral angles of 180° and 60°, respectively. The C30-C191 disulfide bond is also an LH spiral, whereas the C92-C120 disulfide bond is a right-handed (RH) staple. Of the 20 different disulfide bond geometries, LH spirals are the most common and energetically favorable disulfide conformation, with the lowest dihedral strain energy (DSE) (21). The calculated DSE is 9.2 ± 1.1 kJ/mol for each of the two LH spirals and 14.9 ± 1.2 kJ/mol for the RH staple (*SI Appendix*, Table S3) (22, 23).

The structure of g3873 (Fig. 1 *A* and *C*) is dissimilar from all proteins in the PDB (24), as determined by DALI (Z-score > 8) (25) and FoldSeek (E-value threshold = 0.001, sensitivity = 9.5) (26) (Dataset S1 and *SI Appendix*, *Supplementary Methods*). Manual inspection revealed, however, that each set of four clustered alpha helices is reminiscent of the saposin fold (27–29). Saposins are conserved proteins that contain a hydrophobic core sandwiched between two pairs of alpha helices, which allows them to bind phospholipids (30). Although the two four-helical bundles of G3873 resemble the canonical saposin fold, they appear to lack a hydrophobic internal surface that can adopt an alternate open conformation (31).

### A Bicycle Protein with Tandem Saposin-Like Folds.

The second bicycle protein we crystallized, g2703, has a highly divergent amino acid sequence from other bicycle proteins, with no cysteines, but it has a similar intron–exon structure to other *bicycle* genes and was identified as a member of the bicycle gene family using a gene structure classifier (18) (*SI Appendix*, Fig. S4). This protein has an N-terminal secretion signal and is highly expressed in the salivary glands of *H. cornu* (*SI Appendix*, Fig. S5), supporting the inference that it serves as an effector protein. Thus, while *bicycle* genes were named originally based on the presence of conserved C-Y-C motifs, the sequence of g2703 suggests that at least some *bicycle* genes do not fit neatly within this sequence-based categorization.

The X-ray structure of the g2703 protein (Fig. 1 *B* and *D*) was solved at 1.4 Å using selenomethionine-labeled protein (*SI Appendix*, Fig. S1) and multiwavelength anomalous diffraction (MAD) phasing. The structure of g2703 contains two saposin-like domains in tandem (Fig. 1*D*), connected by a long alpha helix. This long central helix appears to have evolved by a merger of the last helix of the first saposin-like unit and the first helix of the second unit (*SI Appendix*, Fig. S6). Similar to g3873, neither of the two saposin-like folds exhibits strong amphipathic properties that might facilitate protein–lipid interaction (32).

The saposin-like domains of g3873 and g2703 share limited structural similarity (33, 34) with the 52 saposin structures deposited in the PDB (24) (*SI Appendix*, Figs. S7 and S8 and Datasets S2 and S3). Most similar to g3873 is the saposin domain of a proteolytically processed human acyloxyacyl hydroxylase saposin (35) (PDB ID: 5W78, TM_score = 0.44 Å, backbone RMSD for 68 residues = 3.65Å) (*SI Appendix*, Fig. S7). The N-terminal saposin-like domain of g2703 showed modest similarity to the saposin domain of prophytepsin (36) from barley, characterized as a vacuolar aspartic proteinase (PDB ID: 1QDM, TM_score = 0.495, backbone RMSD for 71 residues = 4.03 Å) (*SI Appendix*, Fig. S9).

The presence of saposin-like domains in the crystal structures of both g3873 and g2703 suggests that they evolved from a common ancestral protein, despite the absence of significant sequence similarity between these two proteins. This observation provides independent support for the hypothesis that the bicycle gene-structure classifier (18) can identify *bicycle* homologs that cannot be recognized by sequence similarity.

### AlphaFold2 Can Predict Bicycle Protein Structures Using Custom MSA.

**Fig. 2.**
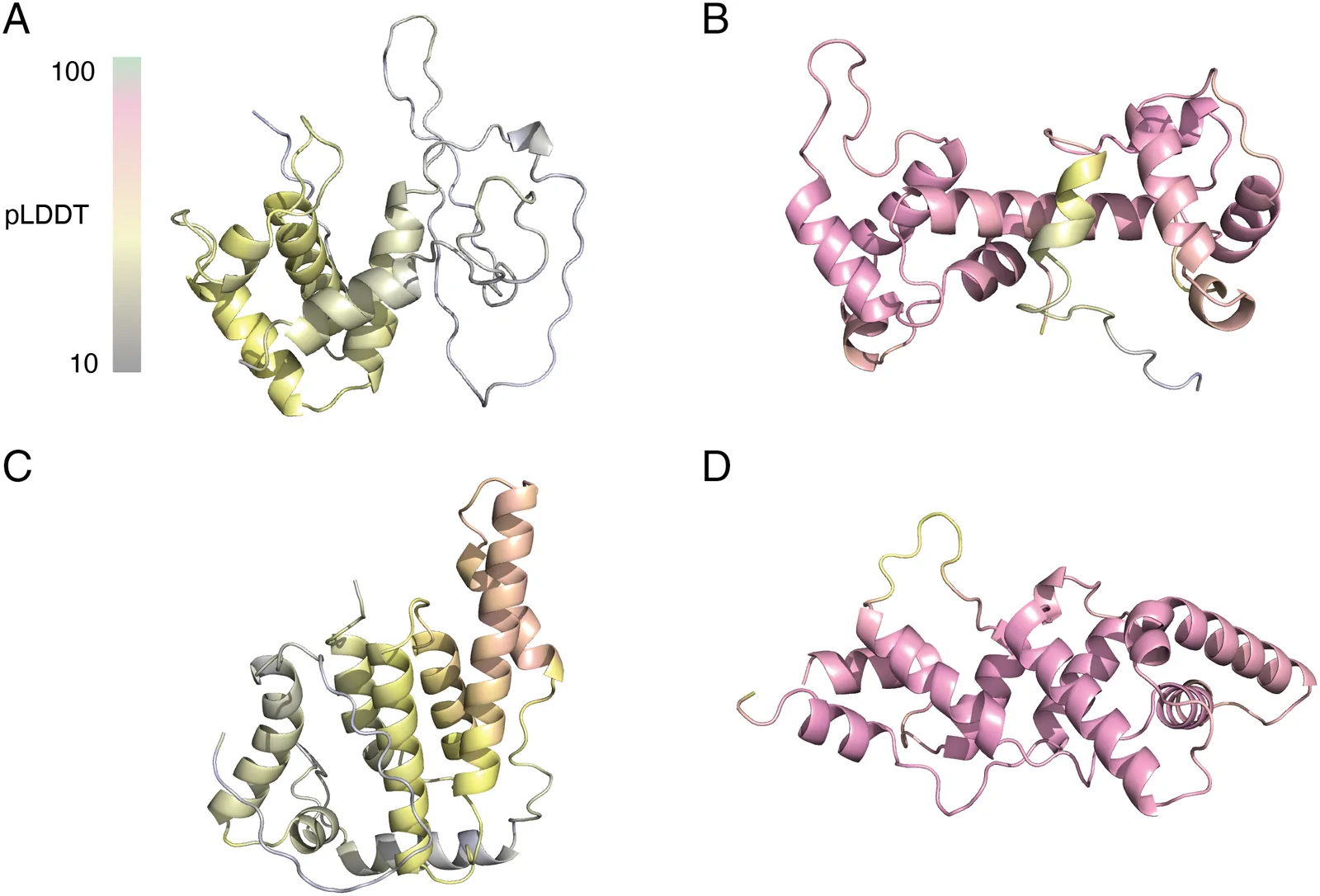
AF2 predicted models for G2703 (*A* and *B*) and G3873 (*C* and *D*). In (*A* and *C*), no MSAs were used because the default databases used by AF2 for homolog searches do not contain any homologs. In (*B* and *D*) custom MSAs were generated with homologous proteins from closely related species. The AF2 predicted models are colored by per-residue measures of local confidence (bar) in the pLDDT. Gray and pink represent ~10 and ~85 confidence levels, respectively.

Since these two bicycle proteins exhibited no significant sequence similarity to others in Genbank (37) and the solved structures were different from all structures in the PDB (24), we submitted our structures as targets for the 15th Critical Assessment of Techniques for Protein Structure Prediction (CASP15) challenge (38, 39). CASP is a community-wide experiment held every 2 y that assesses computational methods for determining protein structures from amino acid sequences. At the CASP15 competition, in the single protein and domain-modeling category, 162 groups submitted predictions for g3873 (CASP ID: T1130) and g2703 (CASP ID: T1131). Final metric GDT_TS scores were used to evaluate the accuracy of the predictions relative to the experimental structures (40). As expected, given the breakthrough success of AlphaFold2 (AF2) in CASP14 (41, 42), most prediction teams deployed deep learning-based algorithms based on AF2, resulting in >90 GDT_TS scores for most targets. The two groups that accurately predicted the structure of g3873 (T1130) (GDT_TS scores > 50) in CASP15 incorporated MSAs of bicycle proteins reported in one of our earlier studies (18). In contrast, no group correctly predicted g2703 (mean GDT_TS score of 12, range 11.65 to 25.31), making g2703 (CASP ID: T1131) the lowest-scoring target (43). This may be because homologs of g2703 were not present in any previously released genome sequences and thus could not be used to generate MSAs. Consistent with the results from CASP15, we also failed to generate accurate predictions for both g2703 or g3873 using AF2, AF3, and ESM-Fold in-house with publicly available sequence databases (44) (Fig. 2 *A* and *C* and *SI Appendix*, Fig. S10).

Given these observations, we hypothesized that MSAs from homologous proteins are required for accurate reconstruction using current machine learning algorithms for structure prediction. We therefore sequenced and annotated the genomes of three species closely related to *H. cornu*: *H. betulae*, *H. hamamelidis,* and *Hamamelistes spinosus* (*SI Appendix*, Fig. S11*A*). In addition, we reannotated genomes from several aphid species distantly related to *H. cornu* (13), *Schlechtendalia chinensis* (45)*, Tetraneura akinire* (18), and *Acyrthosiphon pisum* (46), guided by publicly available RNA-seq data (www.ncbi.nlm.nih.gov/sra). We predicted *bicycle* genes for all species using a gene-structure classifier (18). Strikingly, we observed 1,044 and 800 *bicycle* genes in *H. spinosus* and *S. chinensis*, respectively, revealing very large numbers of *bicycle* genes in these gall forming species and greatly increasing the number of predicted bicycle proteins that were available for study. Using previously published RNA-seq data for *S. chinensis*, we found that *bicycle* genes were expressed at highest levels in the gall foundress generation (*SI Appendix*, Fig. S11*B*), consistent with the hypothesis that bicycle proteins contribute to gall development in this species, as they do in *H. cornu* (13).

Searching the translated aphid proteomes from these seven species with *phmmer* (47) identified 197 and 11 homologs of g3873 and g2703, respectively. Despite the fact that bicycle proteins were not part of the training set used to parameterize AF2, AF2 accurately predicted the experimentally determined X-ray structures of both proteins when provided with these custom MSAs (Fig. 2 *B* and *D* and *SI Appendix*, Fig. S12).

Given the accuracy of the AF2 predictions for the two experimentally solved bicycle proteins, we explored whether AF2 could generate high confidence predictions for 4,924 bicycle proteins from seven aphid species (*SI Appendix*, Fig. S11 *C*–*F* and Dataset S4). MSA depth was positively correlated with prediction confidence (*SI Appendix*, Fig. S11*F*) and AF2 predicted 2,400 high confidence bicycle protein structures (≥80% of amino acids with pLDDT ≥ 60 over at least 70 residues).

As discussed below, aphid bicycle proteins are predicted to occupy a diverse structural space, so it is unclear whether benchmarking with two X-ray structures provides strong confidence for the full range of predictions. However, several observations suggest that these AF2 predicted structures provide a plausible snapshot of the true diversity of bicycle protein structures. First, we benchmarked AF2 for bicycle proteins with two extremely different experimentally derived structures (g3873 and g2703), providing some confidence that AF2 can predict diverse bicycle protein structures. Second, as discussed further below, all of the high confidence predictions include saposin-like folds. And, third, we focus only on predictions with high average predicted local distance difference test (pLDDT) scores, which has been shown to be a reliable measure of fold accuracy (48).

To explore the robustness of this approach, we examined the effect of MSA depth on AF2 performance. First, we predicted structures using randomly resampled MSAs of g3873 and g2703 (*SI Appendix*, Fig. S13 *A*–*D*). The MSA for g3873 was more informative because of the greater number of homologs available for this protein. Predictions with MSAs of at least 16 homologs generated predictions with similar accuracy to the full MSA of 197 homologs (*SI Appendix*, Fig. S13 *A* and *B*). The analysis of G2703 showed a similar trend (*SI Appendix*, Fig. S13 *C* and *D*). To determine if this result can be generalized, we randomly subsampled MSAs from 100 randomly chosen bicycle proteins with high confidence structure predictions and again observed that accuracy leveled off for predictions using MSAs with at least 16 homologs (*SI Appendix*, Fig. S13 *E* and *F*). Thus, sequencing genomes of clusters of closely related species provides a potentially powerful method to generate informative MSAs for protein structure prediction of rapidly evolving protein families.

### Bicycle Proteins Exhibit Extensive Structural Diversity and Surface Variability.

We used this dataset of 2,400 high confidence bicycle protein predictions to explore four questions: Is the saposin-like fold conserved across aphid bicycle protein structures? Are the tandem and helix-swapped permuted structures related? How conserved is the bicycle protein structural and physicochemical space? Finally, does the conformational space and the associated physicochemical signature of bicycle proteins permit inference of a putative function for bicycle proteins?

#### Saposin-like folds are found in all aphid bicycle proteins.

All high-confidence predicted folds possess at least one saposin-like domain. Nonetheless, these domains could not be perfectly superimposed and instead displayed a wide variety of quantitative variations in saposin-like domain shape, size, and arrangement, as we discuss in more detail below.

#### The tandem-linked bicycle proteins likely evolved from helix-swapped proteins.

Almost all of the bicycle proteins in our database encode proteins with helix-swapped saposin-like domains. We therefore wondered how the tandem-linked bicycle proteins, like g2703, evolved. *Bicycle* genes are often found in large paralog groups, apparently reflecting historical gene duplication and divergence (13). We therefore examined the genomic context near *g2703* and identified four putative paralogs. The proteins encoded by these paralogs share almost no sequence similarity with g2703 (*SI Appendix*, Figs. S4 and S6), putting them in the “twilight zone” of protein similarity (49). Strikingly, these four putative paralogs are predicted to encode proteins with tandem-linked saposin-like domains, despite the predicted presence of disulfide bonds (*SI Appendix*, Figs. S4 and S6). One plausible evolutionary model is, therefore, that most genes in this paralog group first evolved a tandem-linked arrangement with disulfide bonds and g2703 evolved by duplication from these paralogs and subsequent loss of cysteines and most recognizable sequence similarity.

Mapping protein structure and intron–exon structure onto a MSA of these paralogs revealed that the alpha-helical “handle” that connects the two tandem-linked saposin-like domains of g2703 likely evolved through fusion of the last and first helices from the first and second saposin-like domains, respectively (*SI Appendix*, Fig. S6). This MSA also shows gene intron positions and reveals the extreme diversity of exon number and protein sequence, even between these genes from a single paralog family. It is striking that *g2703* is one of the most highly expressed *bicycle* genes in *H. cornu* salivary glands (*SI Appendix*, Fig. S5), implying that despite its relatively recent origin and highly divergent sequence, it has acquired an important function for aphid biology during gall development.

#### Bicycle proteins exhibit diverse sequences and structures.

**Fig. 3.**
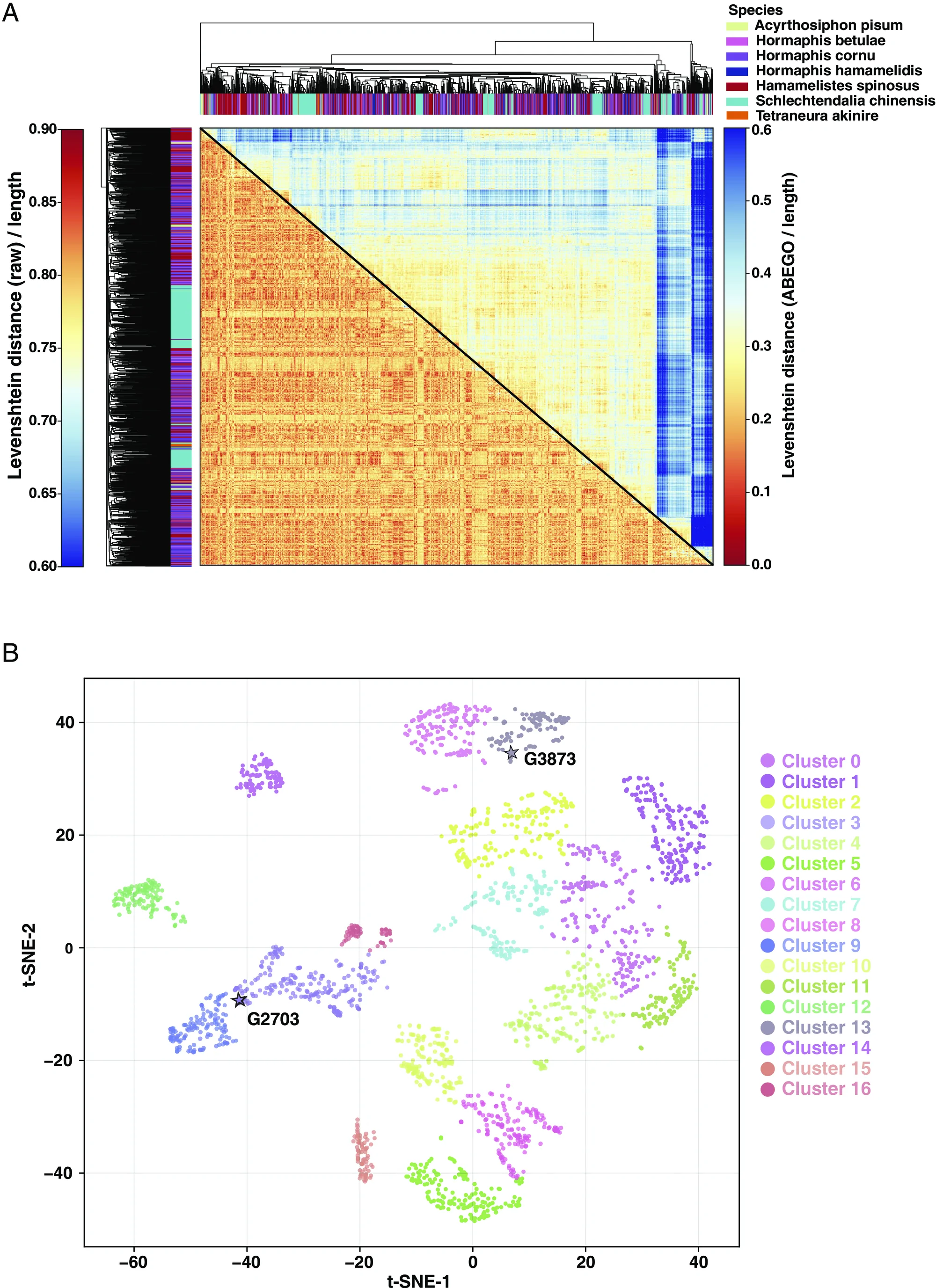
Bicycle proteins are predicted to adopt a wide diversity of structures. (*A*) Heatmap of pairwise normalized Levenshtein distances for 2,400 bicycle proteins from seven species computed using amino acid sequences (*Lower*) and ABEGO-encoded backbone-state strings derived from each AF2-predicted model (*Upper*). In both heatmaps, colors represent distance (0 = identical strings; 1 = maximally different, normalized to the length). Bicycle amino acid sequences display extreme diversity (heatmap is colored blue to red, ranging from 0.6 to 1.0), even within species, whereas ABEGO strings retain intermediate similarity (heatmap distances colored from blue to red from 0.0 to 0.6), even across species. These patterns suggest that bicycle proteins exhibit some conserved higher-order backbone structural grammar despite low primary sequence identity. The dendrograms were calculated by hierarchical clustering the distance measures shown in the heatmaps. Dendrogram tips are colored by species (see key), revealing that amino acid sequences and protein structures are divergent across all aphid species sampled. (*B*) t-SNE embedding of all-vs.-all TM scores for 2,400 bicycle proteins from seven species. Each point represents one protein and is colored according to its cluster assignment (k_cluster_ = 17). Structurally similar proteins (higher TM scores) are located close to each other.

Given this evidence that bicycle proteins can possess diverse structures, we were motivated to further explore bicycle protein diversity. We first characterized protein sequence divergence using pairwise length-normalized Levenshtein distances (50) (lower half below diagonal of Fig. 3*A*). Almost all sequences are highly divergent from each other, with most pairwise distances >0.75. The dendrogram adjacent to the distance matrix (Fig. 3*A*) reveals that some sequences cluster together within species, especially for *S. chinensis*, but even these sequences do not exhibit clear clusters of similarity that might define subfamilies. Thus, bicycle proteins display little evidence of sequence similarity, extending our initial observations of the extreme diversity of bicycle protein sequences within species (13, 18).

We then explored AF2 predicted structural diversity by calculating Levenshtein distances using the ABEGO classification (51) of structural motifs of amino acids (upper half of Fig. 3*A*). The ABEGO-based map exhibits a wider dynamic range than the raw amino acid sequence-based map, with distances saturating at approximately 0.5 rather than near 0.8 (Fig. 3*A*), suggesting that bicycle proteins do share similarities in ABEGO space. Also, the ABEGO distance matrix displays some clustering, although little of this clustering is by species (dendrogram on *Top* of Fig. 3*A*).

**Fig. 4.**
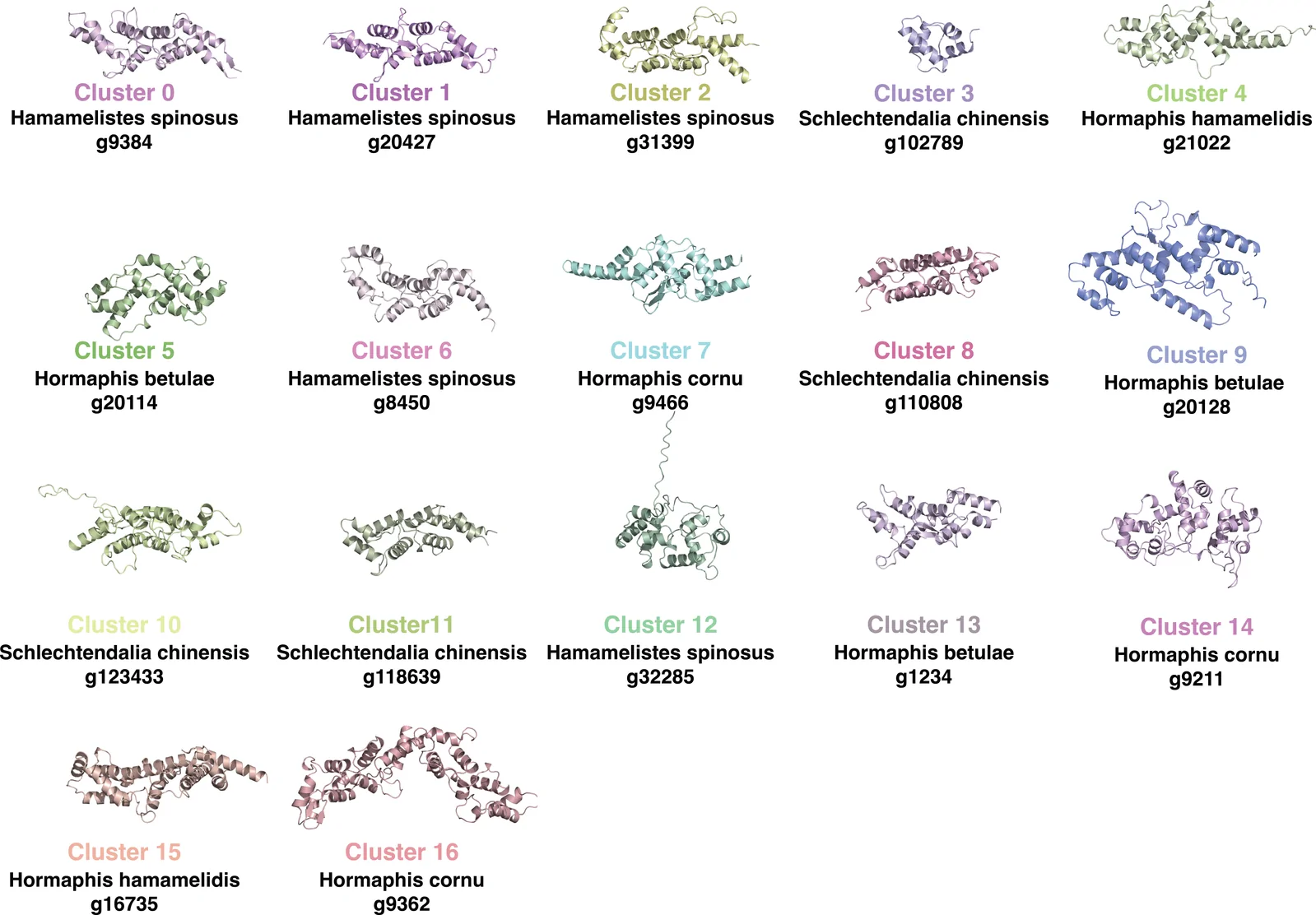
Ribbon models of the medoids representative for each Leiden cluster from Figure 3B shown in their corresponding cluster colors. The medoid possesses the highest average TM-score (the lowest average distance) to all other members of that cluster.

To further explore the clustering observed in the ABEGO-based map, we performed t-SNE (52) embedding of all-vs.-all TM-align comparisons for all 2,400 proteins (Fig. 3*B*). Many distinct structural neighborhoods were identified in this embedding. Medoid models for each cluster illustrate that bicycle proteins occur in a wide variety of arrangements, including one, two, three, four, or six saposin-like domains (Fig. 4). Substantial species partitioning was observed for some clusters (*SI Appendix*, Figs. S14 and S15). This is most striking for the phylogenetically isolated species in our study: *A. pisum*, *T. akinire*, and *S. chinensis*. Nonetheless, bicycle proteins from *Hormaphis* and *Hamamelistes* occupy most of these clusters, indicating that the relative arrangements of the alpha helices in the saposin-like domains vary extensively, even within species. Thus, the bicycle protein family has evolved to occupy a large and discontinuous region of structural space within the context of the saposin-like four-helix domains.

#### Bicycle proteins occupy diverse physicochemical spaces.

**Fig. 5.**
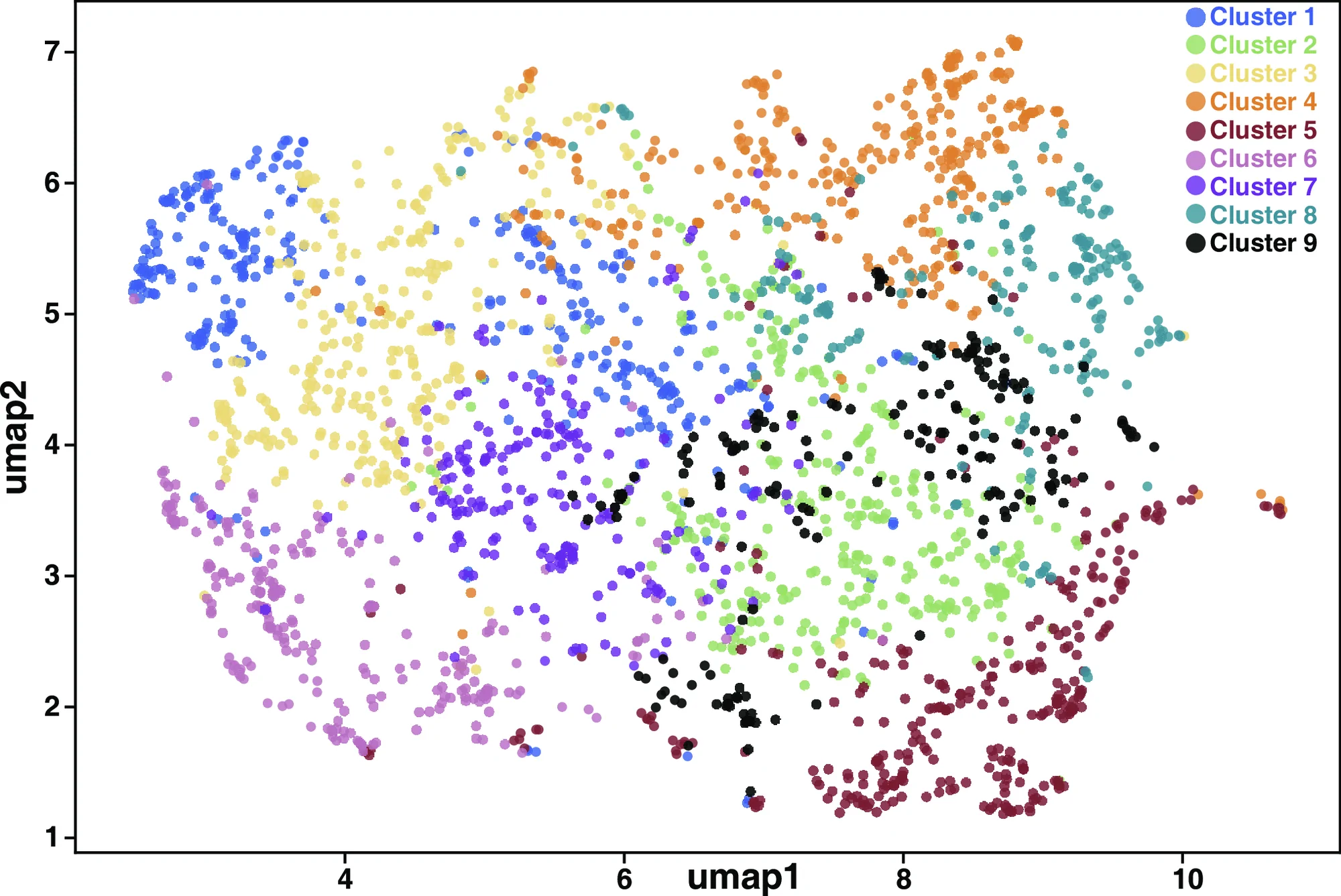
Bicycle proteins display a wide and almost continuous diversity of physicochemical features in the first two dimensions of a UMAP embedding. Each point represents one protein. Different Leiden clusters are shown in different colors and contain sets of proteins primarily with different quantitative combinations of surface hydrophobicity, charge balance, and amphipathicity.

In an effort to identify features that may be conserved among the bicycle proteins, we assessed the physicochemical features of each predicted protein. Five complementary properties were examined: surface chemistry, electrostatic charge, amphipathic character, surface hydrophobic patchiness, and surface texture (*SI Appendix*, Table S4). We embedded these features using UMAP (52) and attempted to identify groups of similar proteins using Leiden clustering (50) (Fig. 5). Even though UMAP tends to “aggressively” cluster points (53), we observed an almost uniform distribution of bicycle proteins characterized by these five properties, suggesting that bicycle proteins sample relatively uniformly across this physicochemical space.

We observed only a few interpretable subgroups in this physicochemical space, defined primarily by distinct combinations of surface hydrophobicity, charge balance, and amphipathicity. One subset of clusters is defined primarily by hydrophobicity and patch morphology: clusters 1, 6, and 3 combine hydrophobicity with minimal amphipathicity. A second subset is dominated by electrostatics and charge composition, being either positively charged (cluster 2), negatively charged (cluster 4), or possessing a pronounced surface dipole (cluster 5). The third subset possesses amphipathic helices and high hydrophobic moment (clusters 7 and 9) or low hydrophobicity with amphipathic signatures (cluster 8). Poisson–Boltzmann surface electrostatics were superimposed on the mediod structures of each cluster to aid visualization of these physicochemical subtypes (*SI Appendix*, Fig. S16). The physicochemical subtypes are not species-partitioned (*SI Appendix*, Figs. S17–S19), suggesting that the variability in physicochemical features extends across the bicycle protein family.

#### Lack of conserved domains thwarts the prediction of bicycle protein functions.

The above analysis suggests that there is limited conservation of features that might provide clues about the molecular function of bicycle proteins. Therefore, we examined whether surface residues displayed any patterns of conservation. We mapped Shannon entropy of residues derived from a MSA of bicycle proteins, which provides a quantitative measure of per-residue conservation, onto the crystal structure of g3873 (*SI Appendix*, Fig. S20). All conserved residues are buried within the interior of the protein, likely required for structural packing, whereas all surface residues show extreme variability.

To assess whether bicycle proteins share structural similarities with known cysteine-rich insect protein families, the disulfide connectivity of all predicted bicycle protein models from seven aphid species was classified (*SI Appendix*, Fig. S21). Three dominant Cys-connectivity patterns were identified connecting C1-C4, C2-C3; C1-C6, C2-C3, C4-C5; and C1-C5, C3-C4 with a free C2), together accounting for ~28% of the dataset. None match the interlocking C1-C3, C2-C5, C4-C6 topology of classic odorant-binding proteins (OBPs) (54), nor the Plus-C or Atypical variants, ruling out OBP family membership.

To examine the energetic landscape underlying this conservation pattern, MSA-level frustration analysis (FrustraEvo) was performed on the three dominant topology subgroups (*SI Appendix*, Fig. S22). The conserved energetic scaffold consists exclusively of the disulfide staples and a small hydrophobic or aromatic core, with all other positions energetically neutral. Per-residue frustration vs. solvent accessibility further showed that maximally frustrated residues are approximately threefold enriched in the buried decile (~60%) compared with the solvent-exposed decile, opposite to the surface-frustration repatterning reported for fungal orphan candidate effectors (55). Surface residues remain predominantly neutral, consistent with the variability observed in the sequence entropy analysis. This absence of any specific conserved sequence signature on the exterior of the protein precludes an inference of bicycle protein functions from these data.

## Discussion

Pathogens frequently introduce effector proteins into hosts for multiple purposes, including driving physiological and developmental programs beneficial to the parasite, securing access to nutrients, and suppressing the host immune response (56, 57). These proteins often alter host cell structure, gene expression, or normal signaling pathways. Many effector proteins do not exhibit domains of known function, and the putative functions of most effector proteins are challenging to decipher (12, 58, 59). Protein structure can provide one path toward insight into effector functions.

Here we report the crystal structures of two *H. cornu* aphid effector proteins that belong to the bicycle protein family. The CYC motifs present in the g3873 structure contribute to two saposin-like domains, in a helix swapped arrangement, secured by disulfide linkages. In contrast, the g2703 structure includes tandem-linked saposin-like folds. It is striking that although g2703 includes no cysteines, and thus no CYC motifs, it also possesses saposin-like domains. This related structural arrangement suggests that g3873 and g2703 shared a common ancestor that contained saposin-like domains.

Despite the presence of saposin-like domains in these proteins, current evidence suggests that bicycle proteins do not possess functions similar to canonical saposins. Saposins are found across eukaryotes and participate in membrane recycling (27, 31). These proteins contain a strongly hydrophobic core that can be exposed to capture lipids. The structures of the bicycle proteins solved in this study do not display features that would allow a hydrophobic core to be exposed. Instead, the saposin-like fold apparently provides a rigid backbone for a rapidly evolving solvent-exposed surface. Other proteins without sequence similarity to saposins contain domain-swapped saposin-like folds (60), although in this case, pairs of helices are swapped, which is different from the single helix swap observed in g3873. Saposin-like folds have also been observed for other proteins, distinct from those that possess lipid or membrane-modifying properties (61, 62). Thus, the saposin-like fold is likely an example of convergent evolution in multiple independent protein families and may provide a stable backbone that allows diversification in sequence space for multiple functions (62). Another example of this kind of convergent evolution is found in the lipocone family of proteins that act in several functional contexts, including regulation of membrane lipid composition, extracellular polysaccharide biosynthesis, and biogenesis of lipopolysaccharides (63).

While AF2 and other deep learning and protein language models failed to accurately predict bicycle protein structures with default settings and existing databases, we found that AF2 can accurately predict these structures when provided with MSAs of homologous proteins identified from the genomes of closely related species. By sequencing several aphid genomes and reannotating previously available genomes to optimize the annotation of *bicycle* genes, we generated 2,400 high-confidence bicycle protein structures across seven aphids species. This large dataset allowed us to explore aphid bicycle protein structure evolution and to search for sequence, structural, and physicochemical patterns that may provide clues about bicycle protein function.

We observed high levels of sequence divergence among the bicycle proteins (Fig. 3*A*), with no clear clusters of similar sequences, even within a single species. In contrast, the predicted structure space, either ABEGO encoded (Fig. 3*A*) or measured by structural similarity with TM scores (Fig. 3*B*), displayed clusters of similar backbone structures (Fig. 4). Individual clusters were mostly not associated with individual species (*SI Appendix*, Figs. S14 and S15), indicating that a broad diversity of protein shapes are encoded in bicycle proteins from individual species.

In contrast to the clusters in shape space, we observed a nearly continuous distribution of bicycle proteins in physicochemical space (Fig. 5), which is also not strongly partitioned by species. This variability is consistent with the lack of sequence conservation, particularly on the surface of bicycle proteins (*SI Appendix*, Fig. S20). There are thus no surface regions of these proteins that exhibit conservation in sequence or physicochemical space, even among subsets of proteins, that might guide inference of the molecular functions of these proteins. It remains unclear how proteins that exhibit such a remarkable diversity of structures and apparent physicochemical properties can accomplish potent and precise modifications of plant development (13).

The proteins within subclusters of the structural space (Fig. 3*B*) do not tend to cluster within the physicochemical property groups shown in Fig. 5, indicating that structure and physicochemical properties have evolved independently within this protein family. As a result, the diversity of protein structures and surface physicochemical properties highlight the challenge of inferring effector protein function strictly from structural data. Nonetheless, the presence of structural and amino acid sequence diversity exhibited by bicycle proteins, combined with our previous finding that *bicycle* genes have evolved in response to strong positive natural selection (13), suggests that individual bicycle proteins may perform distinct functions in plant cells (13) and/or that they have evolved to evade plant defense recognition systems.

Families of rapidly evolving protein families often participate in host–parasite interactions and inferring their structures and functions remains a challenging problem. We sequenced genomes of closely related species to generate informative MSAs, which enabled accurate protein structure prediction. The success of this approach suggests that large sequencing efforts should balance breadth of taxonomic sampling with focused sampling of multiple closely related species to facilitate large-scale prediction of protein structure for rapidly evolving protein families.

## Materials and Methods

### Tests for Protein Expression.

Seven of the eighteen tested bicycle proteins could be expressed in sufficient amounts in either bacterial or Baculovirus-mediated insect cell expression systems for structural studies. Poor expression of the remaining bicycle proteins may have resulted from improper folding and/or degradation of these cysteine-rich proteins.

### Constructs.

A synthetic gene sequence encoding the predicted secreted g2703 protein was codon optimized for expression in *E. coli* and purchased from IDT DNA Technologies. This DNA was inserted between NotI and BamHI restriction sites into the pET51b vector. (MilliporeSigma/Novagen) The final protein construct contains an N-terminal Strep-Tag II and a C-terminal His_10_ tag. The DNA encoding g3873 was purchased from GenScript and inserted into the pET28a vector (MilliporeSigma/Novagen) using Nde1 and Xho1 restriction sites. The resulting protein construct contains an N-terminal His_6_ tag followed by a TEV cleavage site.

### Protein Expression and Purification.

For protein expression, *E. coli* BL21(DE3) was transformed with the pET51 vector encoding *g2703*. Cells were grown for 8 h at 37 °C in 5 mL of LB medium containing 100 µg/mL carbenicillin. This starter culture was added to 50 mL of modified M9 medium, containing 4 g/L ^15^NH_4_Cl and 0.2 g/L glucose as nitrogen and carbon sources, respectively. Cells were grown overnight and diluted to an A_600_ of 0.25 into 1L of fresh modified M9 medium and grown to an A_600_ of 0.8. Expression was induced with 500 µM IPTG overnight at 18 °C. For selenomethionine labeling, 100 mg/L of selenomethionine was added to the culture at an A_600_ of 0.5, cells were grown at 18 °C for 1 h, induced with 500 µM IPTG, and grown further overnight. Cells were harvested by centrifugation at 4,000 g at 4 °C and resuspended in lysis buffer (50 mM NaH_2_PO_4_, pH 8, 250 mM NaCl, 1× EDTA-free protease inhibitor cocktail) and lysed on ice by sonication (for a total process time of 10 min with 5 s on and 10 s off at 50% power). Cell debris was removed by centrifugation at 16,000 g for 45 min at 4 °C, and the Strep-tagged protein was purified over a StrepTrap HP column (Cytiva) in 50 mM NaH_2_PO_4_, pH 8, 250 mM NaCl, pH 8.0 (affinity buffer). The column was washed with 10 volumes of affinity buffer, and the bound protein was eluted in affinity buffer containing 250 mM desthiobiotin. Protein-containing fractions were pooled and concentrated using an Amicon-type centrifugal device with a 10 kDa cutoff filter. 5 mL of the concentrated sample was further purified by size exclusion chromatography (SEC) over a Sephadex 75 column (Cytiva) in SEC buffer (50 mM NaH_2_PO_4_, pH 7, 150 mM NaCl). Following SEC, a final purification step was performed by ion exchange over a MonoQ column with a thirty-column volume gradient from 0 to 0.5 M NaCl in 50 mM NaH_2_PO_4_, pH 7 buffer. Protein fractions at 0.15 M NaCl containing the purified g2703 were pooled.

For protein expression of the His-tagged g3873 protein, Shuffle T7 cells (New England Biolabs) were transformed with the pET28a vector containing the g3873 gene. Cultures were grown as detailed above for g2703 expression. Harvested cells were resuspended in His-affinity buffer (50 mM NaH_2_PO_4_, pH 8, 250 mM NaCl, 30 mM imidazole) with 1× EDTA-free protease inhibitor cocktail and lysed by sonication as described above. Since most of the protein was in the insoluble fraction, the pellet was further sonicated in denaturing affinity buffer (50 mM NaH_2_PO_4_, pH 8, 250 mM NaCl, 30 mM imidazole, 8 M Urea) and the supernatant was clarified by centrifugation. The cleared supernatant was loaded on two tandem 5 mL HisTrap FF columns (Cytiva), and the columns were washed with five column volumes of denaturing affinity buffer. The bound protein was refolded on the column by washing with 100 column volumes of a linear urea gradient from 8 M urea to 0 M urea in the affinity buffer. After a wash with five column volumes of 6% elution buffer (50 mM NaH_2_PO_4_, pH 8, 150 mM NaCl, 500 mM imidazole):94% affinity buffer, the refolded G3873 protein was eluted in 100% elution buffer. The N-terminal His tag was cleaved by adding 10:1 protein: TEV protease and incubation at room temperature for 4 h. The cleaved g3873 protein was passed over a His-trap affinity column to remove the remaining TEV protease and the His-tag fragment. The column flow-through was further purified by size exclusion over a Sephadex 75 column in the SEC buffer. Protein-containing fractions were pooled and concentrated using an Amicon device with 10 kDa cut-off filters. Final protein concentrations were estimated by measuring A_280_ (NanoDrop Instruments), using an extinction coefficient of 15,600 M^−1^ cm^−1^ for g3873 and 49,800 M^−1^ cm^−1^ for g2703. Protein purity and labeling efficiencies were assessed by mass spectrometry. Pure protein samples were flash-frozen in liquid nitrogen and stored at −80 °C until further use.

### Crystallization and Data Collection.

Both g2703 and g3873 proteins were buffer exchanged into 25 mM HEPES buffer at pH 7.0 and used for crystal screening at 4 °C and 20 °C by the hanging drop procedure. Pure protein was mixed with an equal volume of reservoir solution. Crystallization hits obtained from initial screens were optimized using a range of pH, protein concentration, and varying buffer, salt, and precipitant composition. Diffraction-quality crystals were harvested, flash frozen in liquid nitrogen with cryoprotection in 20% (v/v) glycerol containing reservoir solution. Diffraction datasets were collected at the Stanford Synchrotron Radiation Lightsource (SSRL) BL12-2 beamline. The g2703 and the Se-Met g2703 crystals were in the space group of P2_1_2_1_2_1_ and diffracted to 1.4 Å resolution. The crystals of g3873 diffracted to 2.0 Å and were phased using sulfur-based anomalous diffraction. Crystallographic datasets were integrated and scaled using CCPN. Crystal data collection parameters and all structure statistics are summarized in *SI Appendix*, Table S1.

### Genome Sequencing.

High molecular weight genomic DNA was prepared from samples of aphids collected from a single gall for each of the species *Hormaphis hamamelidis*, *Hormaphis betulae*, and *H. spinosus* (Dataset S6) according to the manufacturer’s protocol (CG000145_SamplePrepDemonstratedProtocol -DNAExtractionSingleInsects.pdf, https://support.10xgenomics.com/genome-exome/sample-prep/doc/demonstrated-protocol-dna-extraction-from-single-insects). HMW DNA was quantified using a Qubit fluorometer (Thermo Fisher Scientific, Cat #Q32866) with the Qubit™ dsDNA BR Assay Kit (Thermo Fisher Scientific, Cat #Q32850) and fragment size was assessed by pulsed-field gel electrophoresis (PFGE). The DNA was run on a 1% agarose gel (Seakem Gold Agarose, Lonza, Rockland, ME, Cat #50150) in 0.5× TBE buffer using the BioRad CHEF Mapper system (BioRad, Hercules, CA, Cat #M1703650) for 15 h. The gel was then stained with SYBR Gold dye (Thermo Fisher Scientific, Waltham, MA, Cat #11494). PFGE results indicated that the aphid DNA ranged in size from 50 to 300 kbp.

For all three species, 10× Genomics Chromium linked-read libraries were generated using the Chromium Genome Library Kit & Gel Bead Kit v2 (10× Genomics, San Francisco, CA, Cat #120258), following the manufacturer’s protocol. The Illumina reads were assembled using the Supernova assembler (ver. 2.1.1).

For *H. spinosus*, HiC reads were acquired to facilitate scaffolding of this genome. A single insect was frozen in liquid nitrogen in an Eppendorf tube, ground with a plastic pestle in 1 mL 1% formaldehyde for 20 min. Formaldehyde was quenched by adding 110 μL 1.25 M glycine for 15 min. The sample was spun at maximum speed for 15 min in a tabletop centrifuge. The liquid was removed and replaced with phosphate buffered saline, then spun again. The supernatant was removed, and the sample was stored at −80 °C prior to shipping to Phase Genomics (Seattle, WA) for Hi-C library preparation and sequencing.

We used a 3D-DNA assembly pipeline (64) in “haploid mode” to detect misjoins in the *H. spinosus* Supernova assembly and generate chromosome-scale super scaffolds. HiC alignments for the 3D-DNA pipeline were generated with Juicer v1.6.2 (65) using default settings. The scaffolded assembly was manually reviewed based on inspection of the HiC contact map using Juicebox Assembly Tools (66). The assembly was screened for contamination with BlobTools v1.0.1 (67, 68) using read coverage from the 10× Genomics linked reads and taxonomy information from BLASTN v2.2.31 (69) searches against the National Center for Biotechnology Information (NCBI) nucleotide database. We assessed the quality and completeness of the final assembly using BUSCO v3.0 (70, 71) with the arthropoda_odb9 gene set (72) and by comparing K-mer content of the 10× Genomics linked reads to the assembly with KAT comp v2.3.1 (73).

### RNA-seq.

For all three species, salivary glands were dissected from individual insects by placing insects in phosphate buffered saline and then grasping insects with forceps at the pronotum and anterior abdomen and pulling apart. The salivary glands remain attached to the brain on the anterior portion. The glands were isolated from other tissue using Minutien insect pins, with their tips bent ∼30°, mounted in microdissecting needle holders. Separated glands and the remaining carcass were collected separately into 100 μL of Arcturus PicoPure Extraction buffer. Total RNA was prepared using the Arcturus PicoPure RNA Isolation kit including the optional DNAse step. Barcoded RNASeq libraries were prepared for Illumina NextSeq 550 sequencing (150 bp PE) with a method described previously (74).

#### Annotation of bicycle genes.

The genome assemblies were annotated with RNAseq evidence using BRAKER (75). We then predicted *bicycle* genes using a gene-structure classifier (18) and manually annotated all predicted *bicycle* genes within Apollo (76) using RNAseq evidence collected from salivary glands. GFF files containing BRAKER annotations plus manual annotations are provided as *SI Appendix*.

### Phylogeny Estimation.

An unrooted phylogeny was estimated for the seven species used in this study (*SI Appendix*, Fig. S4*C*) using *getphylo* (77) using all default parameters and starting with FASTA files of all predicted proteins from the genome annotations for all species.

### AlphaFold2 Protein Structure Prediction.

AlphaFold2 (48) was installed on the Janelia Compute Cluster. Initial predictions for G3873 and G2703 were carried out using default sequence databases. Since these predictions did not match the crystal structures, we generated custom MSAs for bicycle proteins using predicted protein sequences for previously published aphid genomes and our aphid genome sequences using a custom bash shell script (*SI Appendix*). We then ran AlphaFold2 with the flag --use_precomputed_msas. These predictions, generated using custom MSAs, had higher confidence and resulted in models that were similar to the crystal structures for both proteins.

We used the above procedure to predict models of all potential bicycle proteins in *H. cornu*, *A. pisum*, and *S. chinensis*. In addition, we also predicted bicycle protein models for all proteins identified by *jackhmmer* search (78) with G2703.

### AF2 Predictions and Filtering.

The initial set of all predicted models was filtered according to the following criteria: 1) low-quality AF2 predictions were removed, using a generous cut-off of median pLDDT score of 60 for at least 80% of the length of the protein sequence, and 2) the polypeptide had to contain at least 70 residues and more than one alpha helix (*SI Appendix*, Fig. S11). From the initial set of 4,093 proteins, applying the requirement of a median pLDDT >60 for at least 80% of the polypeptide length eliminated 1,530 proteins, and the length and helical content filter eliminated another 140 models. The remaining 2,423 protein models were inspected manually, and an additional 20 models were removed since these proteins exhibited >99.0% sequence identity to another bicycle protein; they were essentially duplicates. Details regarding the number of proteins per aphid species predicted by subsequent analyses are provided in Dataset S5.

### Sequence Variability across the Bicycle Proteins.

The variability in overall amino acid sequence space that is sampled by the bicycle proteins was examined using the length-normalized Levenshtein (50) distance. This provides a measure of the difference between two sequences. For each sequence pair, a and b, the Levenshtein distance d(a,b) was computed, which provides the minimum number of substitutions, insertions, and deletions required to transform a into b. Since the dataset includes proteins with up to six CYC motifs, it is essential to normalize this distance by the protein max length:

$$D\left(a,b\right)=\frac{d\left(a,b\right)}{max\left(a,b\right)},$$

where *a* and *b* denote lengths of the two protein sequences in the pair. This results in a symmetric distance between 0 and 1 for sequences of comparable length (with D=0 for identical sequences) and permits us to compare divergence across the dataset. All pairwise distances were computed in an all-vs.-all manner and used for the heatmap visualization. Identification of the most “isolated” sequences within the bicycle proteins was carried out as follow: for each bicycle protein a, the fraction of other bicycle proteins b whose normalized distance from a exceeds a threshold *T* is calculated:

$$\begin{array}{c} {\mathrm{frac}}_{\geq T}(a)=\frac{1}{N-1}\sum_{b\neq a}\;1\{D(a,b)\geq T\}, \end{array}$$

where N is the total number of bicycle proteins and 1{D(a,b)≥T} is the indicator function. Large values of ${\mathrm{frac}}_{\geq T}\left(a\right)$ indicate that a is distant from a large fraction of members in the dataset (i.e., an isolated sequence), whereas smaller values indicate that a is relatively close to most sequences.

We also generated an ABEGO Levenshtein distance map for all 2,403 bicycle proteins in the filtered dataset. Comparing all 2,403 bicycle proteins demonstrates that the ABEGO sequences within the family are highly diverse. We clustered the ABEGO sequences to illustrate sequence variation across the family and the lack of conserved sequence patterns within each aphid species. The dendrogram was colored at the lowest level using the species to which the corresponding bicycle protein belonged (Fig. 3*B*).

### Evaluating Physicochemical Diversity across Bicycle Proteins.

Physicochemical properties of all predicted bicycle models were computed based on the location and nature of each amino acid in the AF2 predicted structures. A total of 22 different features were evaluated (*SI Appendix*, Table S4 and Dataset S5). The solvent accessible surface area (SASA) was estimated using the FreeSASA algorithm (79), where all atom-level SASA were summed per residue to obtain the total SASA. Charged and hydrophobic SASA partitions were derived by defining hydrophobic (I, L, V, F, C, M, A, W, Y, P), positively charged (K, R, and H at pH 7.0), and negatively charged amino acids (D and E). f_SASA hydrophobic_ was calculated as SASA_hydrophobic_/SASA_total_.

In addition, a set of features describing the charge density and net charge was calculated. The fraction of positive, negative, and hydrophobic residues was calculated by dividing individual values by the length of the sequence. Surface hydropathy, surface roughness, hydrophobic patchiness, amphipathic helical fraction, and max hydrophobic moment were also tabulated. All properties and the formulas used to compute them are described in detail in *SI Appendix*, *Supplementary Methods*.

The presence of correlations between these features was evaluated. Correlation will be present since some features contribute to the same overall property. We checked the range of each variable in order to prevent skewed assessment of the overall physicochemical nature of the proteins. The calculated parameters for all 2,400 bicycle proteins are available in Dataset S5.

Using the reduced set of uncorrelated features, our analysis used a custom Python script within the *sklearn* toolkit (66) and the data were plotted using *matplotlib* (67). Nonlinear dimensionality reduction was performed with t-SNE (51) and UMAP (52, 68) for all nonprotein size-dependent parameters, i.e., 11 out of the 22 original and a reduced set of physicochemical parameters (9 out of 11), with a correlation threshold set at 0.85. After assessing various combinations of parameters, we opted to visualize the UMAP projection using 30 neighbors and a distance of 0.05, with a Leiden resolution of clusters at 1.2. This allowed us to preserve the global structure of the map and maximize the consistency of cluster memberships. Medoids representing each of the clusters were identified and used to highlight the physicochemical properties of members in each cluster. The overall distribution of each feature across aphid species and Leiden clusters is provided in *SI Appendix*, Fig. S16.

### Mapping the Diversity of Structural Space across Bicycle Proteins.

Using the 2400 AF2 predicted bicycle protein models, we performed an all-vs.-all comparison of TM scores (69, 70). This all-vs.- all tm-score matrix was reduced to a two-dimensional space using t-SNE (51), with subsequent Leiden clustering (49). This resulted in a global map of predicted bicycle protein structures (provided in Fig. 3*B*). Medoids representative of each cluster were plotted (Fig. 4). Clustering was based solely on structural features, since no cluster overrepresented a particular aphid species or a defined polypeptide chain length.

## Supplementary Material

Appendix 01 (PDF)

Dataset S01 (XLSX)

Dataset S02 (XLSX)

Dataset S03 (XLSX)

Dataset S04 (XLSX)

Dataset S05 (CSV)

Dataset S06 (XLSX)

## Data Availability

Genomes, RNA seq data have been deposited in NCBI in Bioprojects PRJNA1492693 (80), PRJNA1492206, (81), and PRJNA1491490 (82). Genome annotations are available on FigShare (83).

## References

[1] I. Kaloshian, L. L. Walling, Hemipteran and dipteran pests: Effectors and plant host immune regulators. J. Integr. Plant Biol. **58**, 350–361 (2016). 26467026 10.1111/jipb.12438

[2] J. Stuart, Insect effectors and gene-for-gene interactions with host plants. Curr. Opin. Insect Sci. **9**, 56–61 (2015). 32846709 10.1016/j.cois.2015.02.010

[3] C. A. Schneider, E. Calvo, K. E. Peterson, Arboviruses: How saliva impacts the journey from vector to host. Int. J. Mol. Sci. **22**, 9173 (2021). 34502092 10.3390/ijms22179173 PMC8431069

[4] D. Giron, E. Huguet, G. N. Stone, M. J. A. Body, Insect-induced effects on plants and possible effectors used by galling and leaf-mining insects to manipulate their host-plant. J. Insect Physiol. **84**, 70–89 (2016). 26723843 10.1016/j.jinsphys.2015.12.009

[5] M. B. Vogt , Mosquito saliva alone has profound effects on the human immune system. PLoS Negl. Trop. Dis. **12**, e0006439 (2018). 29771921 10.1371/journal.pntd.0006439 PMC5957326

[6] D. A. Lefteri , Mosquito saliva enhances virus infection through sialokinin-dependent vascular leakage. Proc. Natl. Acad. Sci. U.S.A. **119**, e2114309119 (2022). 35675424 10.1073/pnas.2114309119 PMC9214539

[7] P. Li, Y.-J. Lu, H. Chen, B. Day, The lifecycle of the plant immune system. Crit. Rev. Plant Sci. **39**, 72–100 (2020). 33343063 10.1080/07352689.2020.1757829 PMC7748258

[8] M. Vinkler , Understanding the evolution of immune genes in jawed vertebrates. J. Evol. Biol. **36**, 847–873 (2023). 37255207 10.1111/jeb.14181 PMC10247546

[9] G.-Z. Han, Origin and evolution of the plant immune system. New Phytol. **222**, 70–83 (2019). 30575972 10.1111/nph.15596

[10] T. Nyman, F. Bokma, J.-P. Kopelke, Reciprocal diversification in a complex plant-herbivore-parasitoid food web. BMC Biol. **5**, 49–49 (2007). 17976232 10.1186/1741-7007-5-49 PMC2203972

[11] D. J. Obbard, F. M. Jiggins, D. L. Halligan, T. J. Little, Natural selection drives extremely rapid evolution in antiviral RNAi genes. Curr. Biol. **16**, 580–585 (2006). 16546082 10.1016/j.cub.2006.01.065

[12] D. A. Elzinga, G. Jander, The role of protein effectors in plant-aphid interactions. Curr. Opin. Plant Biol. **16**, 451–456 (2013). 23850072 10.1016/j.pbi.2013.06.018

[13] A. Korgaonkar , A novel family of secreted insect proteins linked to plant gall development. Curr. Biol. **31**, 1836–1849.e12 (2021). 33657407 10.1016/j.cub.2021.01.104 PMC8119383

[14] J. C. Schultz, P. P. Edger, M. J. A. Body, H. M. Appel, A galling insect activates plant reproductive programs during gall development. Sci. Rep. **9**, 1833 (2019). 30755671 10.1038/s41598-018-38475-6 PMC6372598

[15] G. N. Stone, K. Schönrogge, The adaptive significance of insect gall morphology. Trends Ecol. Evol. **18**, 512–522 (2003).

[16] M. Rostás, D. Maag, M. Ikegami, M. Inbar, Gall volatiles defend aphids against a browsing mammal. BMC Evol. Biol. **13**, 193 (2013). 24020365 10.1186/1471-2148-13-193 PMC3847210

[17] M. Inbar, A. Eshel, D. Wool, Interspecific competition among phloem-feeding insects mediated by induced host-plant sinks. Ecology **76**, 1506–1515 (1995).

[18] D. L. Stern, C. Han, Gene structure-based homology search identifies highly divergent putative effector gene family. Genome Biol. Evol. **14**, evac069 (2022). 35660862 10.1093/gbe/evac069 PMC9168663

[19] C. K. Riener, G. Kada, H. J. Gruber, Quick measurement of protein sulfhydryls with Ellman’s reagent and with 4, 4′-dithiodipyridine. Anal. Bioanal. Chem. **373**, 266–276 (2002). 12110978 10.1007/s00216-002-1347-2

[20] G. L. Ellman, Tissue sulfhydryl groups. Arch. Biochem. Biophys. **82**, 70–77 (1959). 13650640 10.1016/0003-9861(59)90090-6

[21] J. Richardson, “The anatomy and taxonomy of protein structure” in Advances in Protein Chemistry, C. B. Anfinsen, J. T. Edsall, F. M. Richards, Eds. (Academic Press, 1981), **vol. 34**, pp. 167–339. 10.1016/s0065-3233(08)60520-3 7020376

[22] B. Schmidt, L. Ho, P. J. Hogg, Allosteric disulfide bonds. Biochemistry **45**, 7429–7433 (2006). 16768438 10.1021/bi0603064

[23] P. M. Harrison, M. J. Sternberg, The disulphide beta-cross: From cystine geometry and clustering to classification of small disulphide-rich protein folds. J. Mol. Biol. **264**, 603–623 (1996). 8969308 10.1006/jmbi.1996.0664

[24] H. M. Berman , The protein data bank. Nucleic Acids Res. **28**, 235–242 (2000). 10592235 10.1093/nar/28.1.235 PMC102472

[25] L. Holm, A. Laiho, P. Törönen, M. Salgado, DALI shines a light on remote homologs: One hundred discoveries. Protein Sci. **32**, e4519 (2023). 36419248 10.1002/pro.4519 PMC9793968

[26] M. van Kempen , Fast and accurate protein structure search with Foldseek. Nat. Biotechnol. **42**, 243–246 (2024). 37156916 10.1038/s41587-023-01773-0 PMC10869269

[27] M. Rossmann , Crystal structures of human saposins C and D: Implications for lipid recognition and membrane interactions. Structure **16**, 809–817 (2008). 18462685 10.1016/j.str.2008.02.016

[28] H. Bruhn, A short guided tour through functional and structural features of saposin-like proteins. Biochem. J. **389**, 249–257 (2005). 15992358 10.1042/BJ20050051 PMC1175101

[29] Y. Kishimoto, M. Hiraiwa, J. O’Brien, Saposins: Structure, function, distribution, and molecular genetics. J. Lipid Res. **33**, 1255–1267 (1992). 1402395

[30] E. Liepinsh, M. Andersson, J. M. Ruysschaert, G. Otting, Saposin fold revealed by the NMR structure of NK-lysin. Nat. Struct. Biol. **4**, 793–795 (1997). 9334742 10.1038/nsb1097-793

[31] V. E. Ahn, K. F. Faull, J. P. Whitelegge, A. L. Fluharty, G. G. Privé, Crystal structure of saposin B reveals a dimeric shell for lipid binding. Proc. Natl. Acad. Sci. U.S.A. **100**, 38–43 (2003). 12518053 10.1073/pnas.0136947100 PMC140876

[32] R. Gautier, D. Douguet, B. Antonny, G. Drin, HELIQUEST: A web server to screen sequences with specific alpha-helical properties. Bioinformatics **24**, 2101–2102 (2008). 18662927 10.1093/bioinformatics/btn392

[33] Y. Zhang, J. Skolnick, Scoring function for automated assessment of protein structure template quality. Proteins **57**, 702–710 (2004). 15476259 10.1002/prot.20264

[34] J. Xu, Y. Zhang, How significant is a protein structure similarity with TM-score = 0.5? Bioinformatics **26**, 889–895 (2010). 20164152 10.1093/bioinformatics/btq066 PMC2913670

[35] A. Gorelik, K. Illes, B. Nagar, Crystal structure of the mammalian lipopolysaccharide detoxifier. Proc. Natl. Acad. Sci. U.S.A. **115**, E896–E905 (2018). 29343645 10.1073/pnas.1719834115 PMC5798384

[36] J. Kervinen , Crystal structure of plant aspartic proteinase prophytepsin: Inactivation and vacuolar targeting. EMBO J. **18**, 3947–3955 (1999). 10406799 10.1093/emboj/18.14.3947 PMC1171470

[37] D. A. Benson , GenBank. Nucleic Acids Res. **41**, D36–D42 (2013). 23193287 10.1093/nar/gks1195 PMC3531190

[38] A. Kryshtafovych, T. Schwede, M. Topf, K. Fidelis, J. Moult, Critical assessment of methods of protein structure prediction (CASP)-Round XV. Proteins **91**, 1539–1549 (2023). 37920879 10.1002/prot.26617 PMC10843301

[39] L. T. Alexander , Protein target highlights in CASP15: Analysis of models by structure providers. Proteins **91**, 1571–1599 (2023). 37493353 10.1002/prot.26545 PMC10792529

[40] A. Zemla, Venclovas, J. Moult, K. Fidelis, Processing and evaluation of predictions in CASP4. Proteins **suppl. 5**, 13–21 (2001). 10.1002/prot.10052 11835478

[41] E. Callaway, “It will change everything”: DeepMind’s AI makes gigantic leap in solving protein structures. Nature **588**, 203–204 (2020). 33257889 10.1038/d41586-020-03348-4

[42] J. Jumper , Applying and improving AlphaFold at CASP14. Proteins **89**, 1711–1721 (2021). 34599769 10.1002/prot.26257 PMC9299164

[43] A. Kryshtafovych, T. Schwede, M. Topf, K. Fidelis, J. Moult, Critical assessment of methods of protein structure prediction (CASP)-round XIV. Proteins **89**, 1607–1617 (2021). 34533838 10.1002/prot.26237 PMC8726744

[44] A. J. Simpkin , Tertiary structure assessment at CASP15. Proteins **91**, 1616–1635 (2023). 37746927 10.1002/prot.26593 PMC10792517

[45] H.-Y. Wei , Chromosome-level genome assembly for the horned-gall aphid provides insights into interactions between gall-making insect and its host plant. Ecol. Evol. **12**, e8815 (2022). 35475184 10.1002/ece3.8815 PMC9021935

[46] T. C. Mathers , Chromosome-scale genome assemblies of aphids reveal extensively rearranged autosomes and long-term conservation of the X chromosome. Mol. Biol. Evol. **38**, 856–875 (2021). 32966576 10.1093/molbev/msaa246 PMC7947777

[47] S. R. Eddy, Accelerated profile HMM searches. PLoS Comput. Biol. **7**, e1002195 (2011). 22039361 10.1371/journal.pcbi.1002195 PMC3197634

[48] J. Jumper , Highly accurate protein structure prediction with AlphaFold. Nature **596**, 583–589 (2021). 34265844 10.1038/s41586-021-03819-2 PMC8371605

[49] B. Rost, Twilight zone of protein sequence alignments. Protein Eng. **12**, 85–94 (1999). 10195279 10.1093/protein/12.2.85

[50] V. A. Traag, L. Waltman, N. J. van Eck, From Louvain to Leiden: Guaranteeing well-connected communities. Sci. Rep. **9**, 5233 (2019). 30914743 10.1038/s41598-019-41695-z PMC6435756

[51] K. Sakuma , Design of complicated all-α protein structures. Nat. Struct. Mol. Biol. **31**, 275–282 (2024). 38177681 10.1038/s41594-023-01147-9 PMC11377298

[52] L. van der Maaten, G. Hinton, Visualizing data using t-SNE. J. Mach. Learn. Res. **9**, 2579–2605 (2008).

[53] L. McInnes, J. Healy, J. Melville, UMAP: Uniform manifold approximation and projection for dimension reduction. arXiv [Preprint] (2020). 10.48550/arXiv.1802.03426 (Accessed 15 October 2025).

[54] K. Rihani, J.-F. Ferveur, L. Briand, The 40-year mystery of insect odorant-binding proteins. Biomolecules **11**, 509 (2021). 33808208 10.3390/biom11040509 PMC8067015

[55] M. C. Derbyshire, S. Raffaele, Surface frustration re-patterning underlies the structural landscape and evolvability of fungal orphan candidate effectors. Nat. Commun. **14**, 5244 (2023). 37640704 10.1038/s41467-023-40949-9 PMC10462633

[56] P. N. Dodds, J. P. Rathjen, Plant immunity: Towards an integrated view of plant-pathogen interactions. Nat. Rev. Genet. **11**, 539–548 (2010). 20585331 10.1038/nrg2812

[57] M. C. Giraldo, B. Valent, Filamentous plant pathogen effectors in action. Nat. Rev. Microbiol. **11**, 800–814 (2013). 24129511 10.1038/nrmicro3119

[58] M. Franceschetti , Effectors of filamentous plant pathogens: Commonalities amid diversity. Microbiol. Mol. Biol. Rev. **81**, e00066-16 (2017). 28356329 10.1128/MMBR.00066-16 PMC5485802

[59] K. Seong, K. V. Krasileva, Prediction of effector protein structures from fungal phytopathogens enables evolutionary analyses. Nat. Microbiol. **8**, 174–187 (2023). 36604508 10.1038/s41564-022-01287-6 PMC9816061

[60] B. C. Bryksa , Structure and mechanism of the saposin-like domain of a plant aspartic protease. J. Biol. Chem. **286**, 28265–28275 (2011). 21676875 10.1074/jbc.M111.252619 PMC3151071

[61] B. C. Bornhauser, P.-A. Olsson, D. Lindholm, MSAP is a novel MIR-interacting protein that enhances neurite outgrowth and increases myosin regulatory light chain. J. Biol. Chem. **278**, 35412–35420 (2003). 12826659 10.1074/jbc.M306271200

[62] R. S. Munford, P. O. Sheppard, P. J. O’Hara, Saposin-like proteins (SAPLIP) carry out diverse functions on a common backbone structure. J. Lipid Res. **36**, 1653–1663 (1995). 7595087

[63] A. M. Burroughs, G. G. Nicastro, L. Aravind, The lipocone superfamily, a unifying theme in metabolism of lipids, peptidoglycan and exopolysaccharides, inter-organismal conflicts and immunity. eLife **14**, RP108061 (2025). 40923594 10.7554/eLife.108061 PMC12419801

[64] O. Dudchenko , De novo assembly of the Aedes aegypti genome using Hi-C yields chromosome-length scaffolds. Science **356**, 92–95 (2017). 28336562 10.1126/science.aal3327 PMC5635820

[65] N. C. Durand , Juicer provides a one-click system for analyzing loop-resolution Hi-C experiments. Cell Syst. **3**, 95–98 (2016). 27467249 10.1016/j.cels.2016.07.002 PMC5846465

[66] O. Dudchenko , The Juicebox Assembly Tools module facilitates de novo assembly of mammalian genomes with chromosome-length scaffolds for under $1000. bioRxiv [Preprint] (2018). https://www.biorxiv.org/content/10.1101/254797v1 (Accessed 17 March 2026).

[67] D. R. Laetsch, M. L. Blaxter, BlobTools: Interrogation of genome assemblies. F1000Res **6**, 1287 (2017).

[68] S. Kumar, M. Jones, G. Koutsovoulos, M. Clarke, M. Blaxter, Blobology: Exploring raw genome data for contaminants, symbionts and parasites using taxon-annotated GC-coverage plots. Front. Genet. **4**, 237 (2013). 24348509 10.3389/fgene.2013.00237 PMC3843372

[69] C. Camacho , BLAST+: Architecture and applications. BMC Bioinformatics **10**, 1–9 (2009). 20003500 10.1186/1471-2105-10-421 PMC2803857

[70] F. A. Simao, R. M. Waterhouse, P. Ioannidis, E. V. Kriventseva, E. M. Zdobnov, BUSCO: Assessing genome assembly and annotation completeness with single-copy orthologs. Bioinformatics **31**, 3210–3212 (2015). 26059717 10.1093/bioinformatics/btv351

[71] R. M. Waterhouse , BUSCO applications from quality assessments to gene prediction and phylogenomics. Mol. Biol. Evol. **35**, 543–548 (2018). 29220515 10.1093/molbev/msx319 PMC5850278

[72] E. M. Zdobnov , OrthoDB v9.1: Cataloging evolutionary and functional annotations for animal, fungal, plant, archaeal, bacterial and viral orthologs. Nucleic Acids Res. **45**, D744–D749 (2017). 27899580 10.1093/nar/gkw1119 PMC5210582

[73] D. Mapleson, G. Garcia Accinelli, G. Kettleborough, J. Wright, B. J. Clavijo, KAT: A K-mer analysis toolkit to quality control NGS datasets and genome assemblies. Bioinformatics **33**, 574–576 (2017). 27797770 10.1093/bioinformatics/btw663 PMC5408915

[74] M. S. Cembrowski , Dissociable structural and functional hippocampal outputs via distinct subiculum cell classes. Cell **173**, 1280–1292.e18 (2018). 29681453 10.1016/j.cell.2018.03.031

[75] K. J. Hoff, A. Lomsadze, M. Borodovsky, M. Stanke, Whole-genome annotation with BRAKER. Methods Mol. Biol. **1962**, 65–95 (2019). 31020555 10.1007/978-1-4939-9173-0_5 PMC6635606

[76] N. A. Dunn , Apollo: Democratizing genome annotation. PLoS Comput. Biol. **15**, e1006790 (2019). 30726205 10.1371/journal.pcbi.1006790 PMC6380598

[77] T. J. Booth, S. Shaw, P. Cruz-Morales, T. Weber, getphylo: Rapid and automatic generation of multi-locus phylogenetic trees. BMC Bioinformatics **26**, 21 (2025). 39827349 10.1186/s12859-025-06035-1 PMC11748604

[78] L. S. Johnson, S. R. Eddy, E. Portugaly, Hidden Markov model speed heuristic and iterative HMM search procedure. BMC Bioinformatics **11**, 431 (2010). 20718988 10.1186/1471-2105-11-431 PMC2931519

[79] S. Mitternacht, FreeSASA: An open source C library for solvent accessible surface area calculations. F1000Res **5**, 189 (2016). 26973785 10.12688/f1000research.7931.1 PMC4776673

[80] F. Bhinderwala , Hamamelistes spinosus Genome sequencing and assembly. NCBI BioProject. https://www.ncbi.nlm.nih.gov/bioproject/?term=PRJNA1492693. Deposited 9 July 2026.

[81] F. Bhinderwala , Hormaphis betulae Genome sequencing and assembly. NCBI BioProject. https://www.ncbi.nlm.nih.gov/bioproject/?term=PRJNA1492206. Deposited 8 July 2026.

[82] F. Bhinderwala , Hormaphis hamamelidis. NCBI BioProject. https://www.ncbi.nlm.nih.gov/bioproject/PRJNA1491490/. Deposited 7 July 2026.

[83] D. Stern , Genome annotations for studies of bicycle protein evolution. Figshare. 10.6084/m9.figshare.33000401. Deposited 16 July 2026.

